# Effect of Unavoidable Ion (Ca^2+^) in Pulp on the Dispersion Behavior of Fine Smithsonite

**DOI:** 10.3390/molecules27249026

**Published:** 2022-12-18

**Authors:** Zhongyi Liu, Jie Liu, Yinfei Liao, Chenxi Jin, Zilong Ma

**Affiliations:** 1 National Engineering Research Centre of Coal Preparation and Purification, China University of Mining and Technology, Xuzhou 221116, China; 2 Sinopec Ningbo Engineering Co., Ltd., Ningbo 315103, China; 3 College of Environmental Science and Engineering, Tongji University, Shanghai 200092, China

**Keywords:** smithsonite, fine particles, calcium ions, dispersion

## Abstract

The efficient dispersion of particles is a prerequisite for the efficient flotation of fine smithsonite. However, unavoidable ions (Ca^2+^) in the pulp have become a challenge for the efficient separation of fine smithsonite, due to the high content of pulp and small radius of hydrated ions. Therefore, the dispersion behavior and mechanism of Ca^2+^ action on smithsonite are important for improving the efficiency of smithsonite flotation. In this study, the effects of Ca^2+^ on the dispersion behavior of fine smithsonite were studied using a turbidity test. The results showed that the dispersion behavior of smithsonite was good in the absence of Ca^2+^ at a range of pH = 4–12. However, the measured turbidity values of smithsonite decreased with the addition of calcium ions. In particular, the dispersion behavior of smithsonite became worse at pH > 10. Zeta potential test results showed that the smithsonite’s surface potential shifted positively, and the absolute value of potential decreased in the presence of Ca^2+^. The results of X-ray photoelectron spectroscopy (XPS) and scanning electron microscopy (SEM) analysis showed that calcium ions were adsorbed on the smithsonite surface, which may have caused ion exchange or the generation of calcium hydroxide precipitation leading to particle coalescence behavior. The calculations of solution chemistry and DLVO theory indicated that calcium ions adsorbed on the surface of smithsonite to form Ca(OH)^+^ or precipitation, which reduced the potential energy of interparticle interactions and led to the disruption of dispersion behavior of smithsonite.

## 1. Introduction

Zinc is an important strategic resource for the country, which is widely used in the steel surface coating, automobile, construction, light industry, medicine, battery, and other industries [1]. According to USGS data, identified zinc resources of the world amounted to about 1.9 billion tons in 2021 [2]. The main sources of zinc are zinc sulfide ores and zinc oxide ores. With the depletion of zinc sulfide ores, zinc oxide ores have become the main source of zinc [3,4]. Smithsonite has attracted interest due to its relative ease of selection and high theoretical grade. Consequently, many scholars have studied the sulfide pretreatment of smithsonite and its flotation separation [5,6].

However, the influence of gangue slimes and unavoidable ions is a difficult challenge in the separation of smithsonite from gangue minerals [1,7]. There is a high content of unavoidable ions (Ca^2+^) and they have an obvious effect on the flotation solution environment of smithsonite. Their small hydration ion radius enables them to act on the surface of smithsonite. Many researchers have studied the effect of Ca^2+^ on the flotation of smithsonite [8]. Studies have shown that metal ions species and concentrations have different effects on the floatability of smithsonite and gangue minerals [9]. The conclusions include that Ca^2+^ has inhibitory effects on smithsonite and calcite, and low concentrations of Ca^2+^ have activating effects on quartz [9]. Chen et al. also found that Ca(OH)_2_(s) and CaOH^+^ were produced due to Ca^2+^ hydrolysis and chemical reactions at pH = 9.5; these preferentially adsorbed on the surface of smithsonite and prevented the adsorption of S^2−^. Sodium carbonate can eliminate the adverse effect of Ca^2+^ on the sulfide flotation of smithsonite [10]. The relevant literature showed that Fe^3+^ hindered the adsorption of xanthate on the surface of smithsonite, resulting in poor floatability [11]. Other studies concluded that Al^3+^ hydrolysis to generate Al(OH)_3_ and Al_2_O_3_ was selectively adsorbed on the calcite surface and hindered the adsorption of collectors, which achieved the flotation separation of smithsonite from calcite [12]. In addition, some studies [13,14] found that the components of inhibition and activation produced by Ca^2+^ are metal hydroxyl complexes and hydroxide precipitation.

It was found that metal ions also affect the dispersion and condensation behavior of minerals [15,16]. However, the effect of calcium ions on the dispersion behavior of fine smithsonite is less studied. It has been shown that metal ions change the surface properties of minerals, leading to the homogeneous or heterogeneous coalescence of particles, which also leads to poor dispersion [17,18]. Meanwhile, fine smithsonite is more able to form coalescence because of its large surface area and strong particle activity. Metal ions can also adsorb on the surface of smithsonite, reducing the action effective area of the collectors, which could deteriorate the flotation environment [19,20,21]. Therefore, it is necessary to investigate the effect of Ca^2+^ on the dispersion behavior of fine smithsonite to improve the flotation efficiency of smithsonite.

In this study, the effect of Ca^2+^ on the dispersion behavior of smithsonite was investigated using a turbidity test. Analyses of Zeta potential, scanning electron microscopy (SEM), energy dispersive spectrometer (EDS), X-ray photoelectron spectrometer (XPS), and calculations of solution chemistry and Derjaguin–Landau–Verwee–Overbeek (DLVO) were used to reveal the mechanism of Ca^2+^ on the dispersion of smithsonite, which could provide a theoretical basis for the efficient separation of smithsonite.

## 2. Results and Discussion

### 2.1. Turbidity Analysis

**Figure 1:**
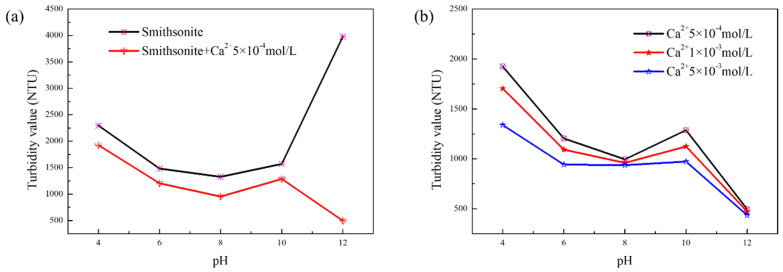
The measured turbidity value of smithsonite: (**a**) smithsonite and smithsonite with Ca^2+^ (5 × 10^−4^ mol/L); (**b**) different concentrations of Ca^2+^.

The measured turbidity value of smithsonite decreased and then increased, as shown in Figure 1a, in the absence of metal ions at pH = 4–12. The dispersion behavior of smithsonite was better in the test pH range. However, the dispersion behavior of smithsonite was affected by the action of calcium ions. The experimental results showed that the changing trend of smithsonite dispersion behavior with Ca^2+^ remained consistent with that without Ca^2+^ in the pH = 4–10 range. However, the smithsonite particles formed flocs and appeared to agglomerate at pH > 10. The measured turbidity value of the smithsonite decreased from 3979 NTU to 496 NTU at pH = 12. It may be that the hydrolysis of Ca^2+^ to produce hydroxyl complexes or hydroxides changes the aggregation and dispersion behavior of mineral particles [22]. The dispersion behavior of smithsonite, as shown in Figure 1b, was different with different Ca^2+^ concentrations. When the concentrations were 5 × 10^−4^ mol/L and 1 × 10^−3^ mol/L, the measured turbidity values did not change much. However, with a concentration of calcium ions of 5 × 10^−3^ mol/L, the turbidity values changed more significantly. The aggregation became more and more obvious with the increase of calcium ion concentration.

### 2.2. Zeta Analysis

**Figure 2:**
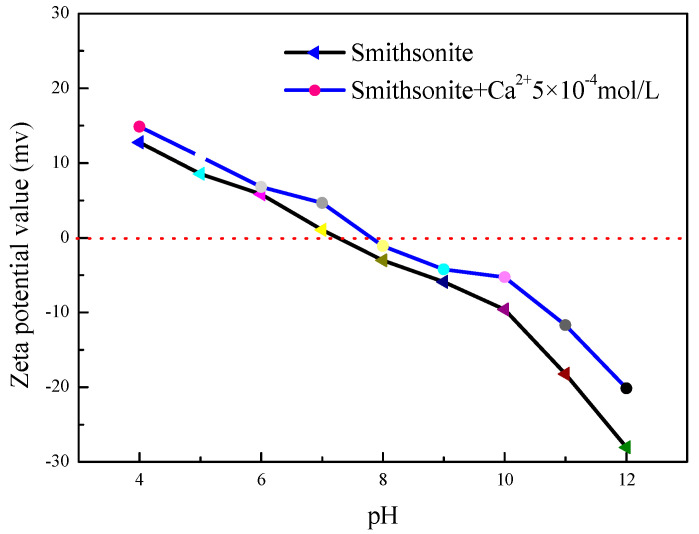
Zeta potential of smithsonite particles in the presence of Ca^2+^ (5 × 10^−4^ mol/L).

The isoelectric point (IEP) of the smithsonite surface in Figure 2 was about pH = 7.2 in deionized distilled water, which was consistent with the results of the relevant literature [23]. This indicates that coalescence of the smithsonite particles may have occurred at about pH = 7.2. At pH < 7.2, the surface charge of smithsonite was positive, which indicates that the dispersion behavior of smithsonite was good. However, there was a reversed result at pH > 7.2. When the pH value increased, the surface electronegativity became stronger. This indicates that the electrostatic force between smithsonite particles was increased.

It can be seen from Figure 2 that there was no change in smithsonite’s surface potential positivity and negativity in the presence of Ca^2+^. In addition, the smithsonite’s surface potential shifted positively and the absolute value of potential became smaller at different pH values. This is due to the calcium ions acting as a compressed double electric layer [24]. There was a rising span of smithsonite surface potential values between pH = 9–12, which may be caused by the adsorption of Ca(OH)^+^ on the surface of the smithsonite, leading to the rise in potential value. When pH = 8–12, the surface potential electronegativity of smithsonite increased after the action of calcium ions, which indicates that interparticle electrostatic repulsion was increased. However, the coalescence behavior of smithsonite particles suggests that electrostatic repulsion was not the dominant factor under alkaline conditions.

### 2.3. Solution Chemistry Analysis

In the saturated state, the dissolution of smithsonite is in the following equilibrium [25].

(1) $${\mathrm{ZnCO}}_{3}\left(s\right)⇌{\mathrm{Zn}}^{2+}{+\mathrm{CO}}_{3}^{2+}{\;K}_{{\mathrm{sp},\mathrm{ZnCO}}_{3}}{=10}^{-9.7}\;$$

(2) $${\mathrm{Zn}}^{2+}{+\mathrm{CO}}_{3}^{2-}⇌{\mathrm{ZnCO}}_{3}\left(\mathrm{aq}\right){\;K}_{1}{=10}^{5.3}\;$$

(3) $${\mathrm{Zn}}^{2+}{+\mathrm{HCO}}_{3}^{2-}⇌{\mathrm{ZnCO}}_{3}^{+}{\;K}_{2}{=10}^{2.1}\;$$

(4) $${\mathrm{Zn}}^{2+}{+\mathrm{OH}}^{-}⇌{\mathrm{ZnOH}}^{+}{\;\beta }_{1}{=10}^{6.5}\;$$

(5) $${\mathrm{Zn}}^{2+}{+2\mathrm{OH}}^{-}⇌{\mathrm{Zn}(\mathrm{OH})}_{2}\left(\mathrm{aq}\right){\;\beta }_{2}{=10}^{\;11.10}\;$$

(6) $${\mathrm{Zn}}^{2+}{+3\mathrm{OH}}^{-}⇌{\mathrm{ZnOH}}_{3}^{-}{\;\beta }_{3}{=10}^{14.31}\;$$

(7) $${\mathrm{Zn}}^{2+}{+4\mathrm{OH}}^{--}⇌{\mathrm{ZnOH}}_{4}^{2-}{\;\beta }_{4}{=10}^{17.70}$$

(8) $$\mathrm{Zn}{\left(\mathrm{OH}\right)}_{2}\left(s\right)⇌{\mathrm{Zn}}^{2+}{+2\mathrm{OH}}^{-}{\;K}_{\mathrm{sp},\mathrm{Zn}{\left(\mathrm{OH}\right)}_{2}}{=10}^{-10.07}\;$$

(9) $$H^{+}{+\mathrm{CO}}_{3}^{2-}⇌{\mathrm{HCO}}_{3}^{-}{\;K}_{1}^{H}{=10}^{10.33}$$

(10) $$H^{+}{+\mathrm{HCO}}_{3}^{-}⇌H_{2}{\mathrm{CO}}_{3}{\;K}_{2}^{H}{=10}^{6.35}\;$$

(11) $$H_{2}{\mathrm{CO}}_{3}⇌{\mathrm{CO}}_{2}\left(g\right){+H}_{2}{O\;K}_{0\;}{=10}^{1.47}$$

In the atmosphere, $P_{{\mathrm{CO}}_{2}}{=10}^{-3.5}\mathrm{atm}$, then $\left[H_{2}{\mathrm{CO}}_{3}\right]{=P}_{{\mathrm{CO}}_{2}}/K_{0}{=10}^{-4.97}$, $\log\left(H_{2}{\mathrm{CO}}_{3}\right)=-4.97$, and the relationship between the concentration of smithsonite and pH can be obtained.

**Figure 3:**
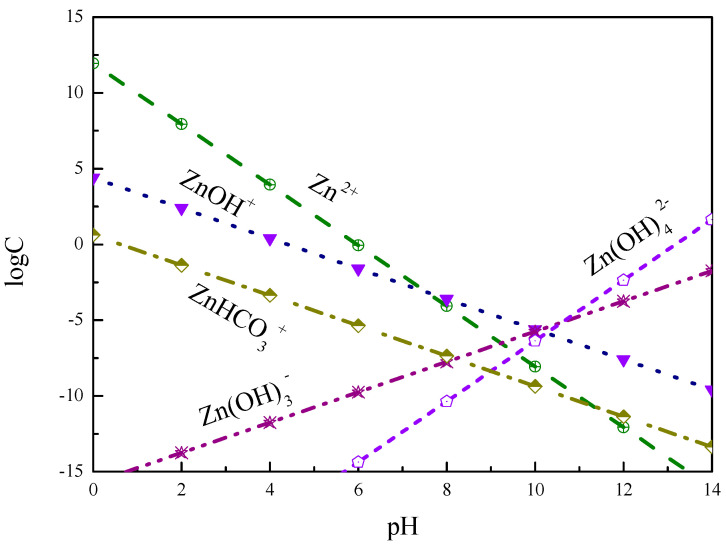
The log C-pH of dissolved compositions of smithsonite in solution.

Figure 3 shows that the dissolved compositions of smithsonite are mainly ZnHCO_3_^+^, Zn^2+^, and ZnOH^+^, which may be positioning ions of smithsonite under acidic conditions. At this time, the dispersion behavior of smithsonite is better because the potential is positive and the interparticle force is mainly electrostatic repulsion. The ZnHCO_3_^+^, Zn^2+^, and ZnOH^+^ compositions gradually decreased with the increase in pH value. Under alkaline conditions, the main ions are Zn(OH)_3_^−^ and Zn(OH)_4_^2−^, and the dispersion behavior of smithsonite is good because the potential value is negative and the interparticle force is mainly electrostatic repulsion.

Table 1 shows the stability constants of Ca^2+^ hydroxyl complexes, and a plot between each composition of metal ions in solution and log C-pH was obtained by calculation.

**Figure 4:**
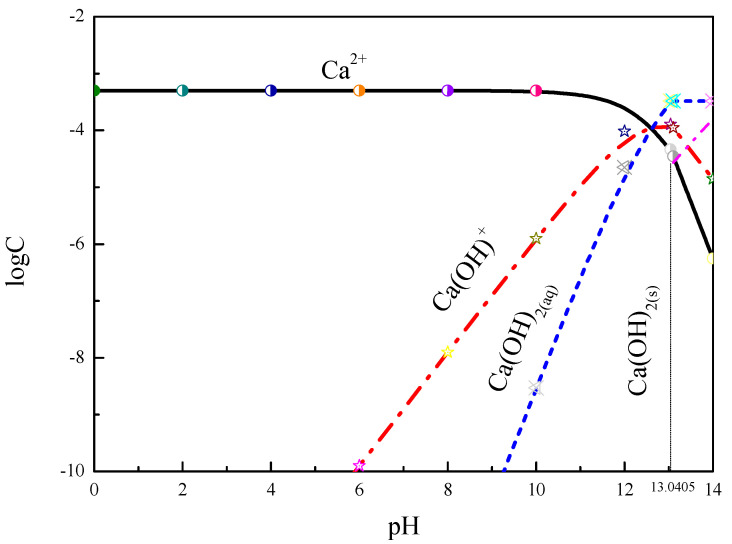
The log C-pH of hydrolytic compositions of Ca^2+^ (5 × 10^−4^ mol/L) in solution.

It can be seen from Figure 4 that the main composition is Ca^2+^ in the test pH range. The composition of Ca(OH)^+^ increased with increasing pH, reaching a high value at pH > 10. The Ca(OH)_2_(aq) content increased with increasing pH, while the Ca^2+^ and Ca(OH)^+^ compositions gradually decreased at pH > 12. The measured turbidity value of smithsonite slightly decreased under acidic conditions, as shown by the turbidity test. The smithsonite particles coalesced severely at pH > 10. It is suggested that Ca(OH)^+^ and Ca(OH)_2(aq)_ are the main compositions promoting the coalescence of smithsonite particles [13].

### 2.4. SEM, EDS, and XPS Analysis

**Figure 5:**
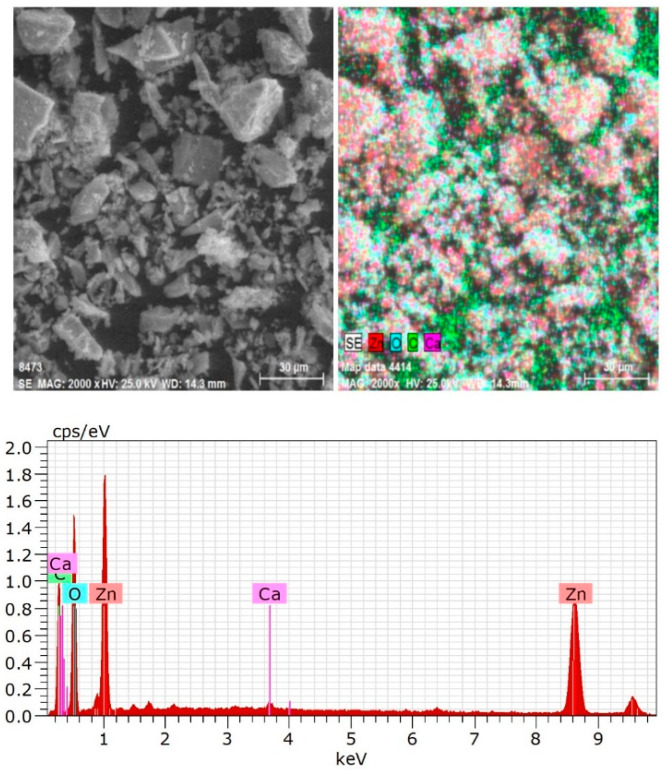
The analysis result of SEM and EDS on smithsonite with Ca^2+^.

It can be seen from Figure 5 that the flocs size of smithsonite can reach above 30 μm in the presence of Ca^2+^. This fully illustrates the results of the turbidity test. From the analysis of the EDS energy spectrum, it can be seen that there were Ca peaks in this spectrum, indicating that Ca^2+^ was adsorbed on the surface of smithsonite.

**Figure 6:**
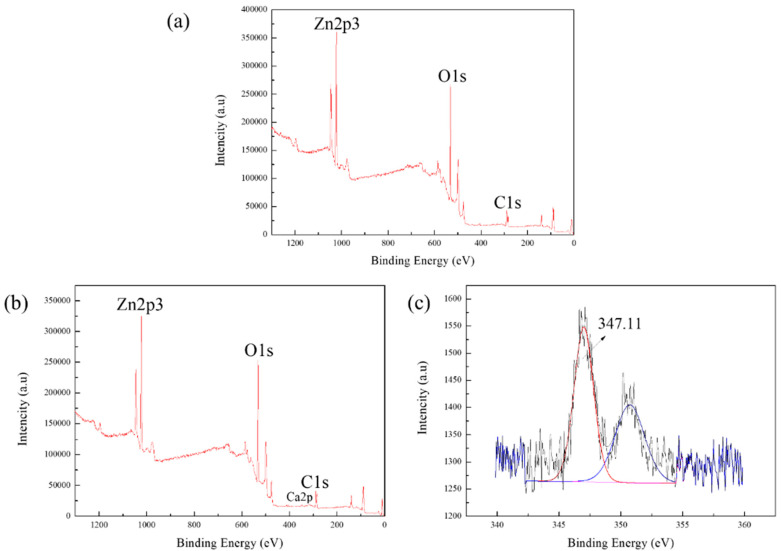
The analysis result of XPS: (**a**) smithsonite (**b**) smithsonite with Ca^2+^ (**c**) Ca2p spectra of the smithsonite surface after action of Ca^2+^.

Figure 6a shows that the XPS spectrum of smithsonite has only three elemental peaks of Zn2p3, C1s, and O1s without Ca^2+^. This indicates that there are no impurities on the smithsonite’s surface, and the analytical results were consistent with the above XRF results. It can be seen from Figure 6b that the XPS analysis spectrum respectively had more Ca2p peaks than in Figure 6a after the addition of Ca^2+^. The atomic concentration of Ca2p on the surface of smithsonite was 0.60% after the action of calcium ions, as shown in Table 2. The results of the fitted narrow-peak profiles of Ca^2+^ also indicated that Ca^2+^ is adsorbed on the surface of the smithsonite. As can be seen in Figure 6c, the curve fit revealed two peaks of Ca2p at 347.11 eV and 350.87 eV, which are similar to Ca2p_3/2_ and Ca2p_1/2_ in calcite [26]. It can be seen from Table 2 that the atomic concentrations of Zn2p, O1s, and C1s were 17.02%, 52.73%, and 30.24%, respectively. However, the results of calcium ion action on smithsonite showed that the atomic concentrations of Zn2p and O1s decreased by 2.09%, and 1.46%, respectively, indicating a decrease in zinc carbonate. This may be due to the ion exchange of Ca^2+^ with the smithsonite lattice ion (Zn^2+^) to form CaCO_3_, or to the hydrolysis of calcium ions adsorbed on the surface of smithsonite to form precipitate [13,27].

### 2.5. Calculation of Surface Energy of Particles Using Classic DLVO Theory

**Figure 7:**
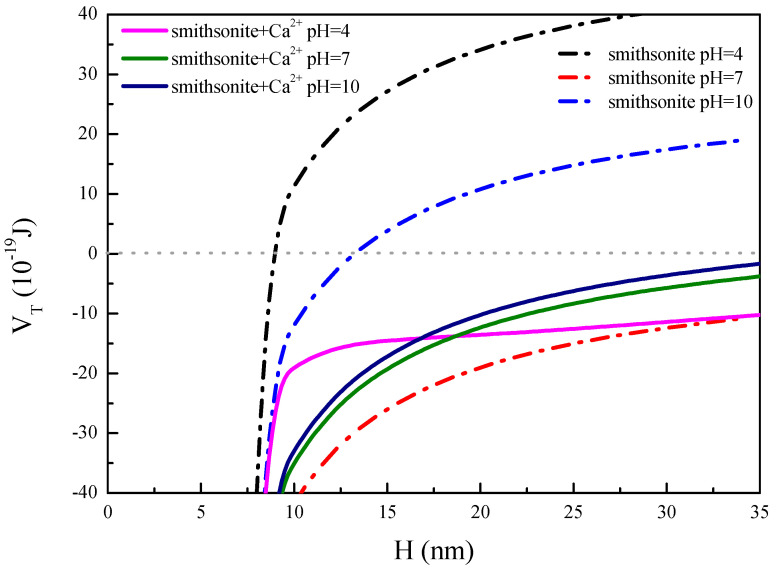
The interaction between smithsonite particles before and after the addition of Ca^2+^ (5 × 10^−4^ mol/L).

According to Equations (12)–(14), the total force between smithsonite particles at pH = 4, pH = 7, and pH = 10 can be calculated, and the results are shown in Figure 7.

Smithsonite particles at three different pH conditions have different potential energy values without Ca^2+^, as shown in Figure 7. The potential energy values of smithsonite are pH = 4 > pH = 10 > pH = 7. When H < 8 nm, the interparticle potential energy is negative at pH = 4 because the van der Waals forces at this distance are dominant forces. When H > 8 nm, the barrier between particles is about 15.54 × 10^−19^ J with the increase of electrostatic repulsion. When H < 13 nm, the interparticle interaction potential energy of smithsonite particles is negative at pH = 10. When H > 13 nm, the barrier between particles is about 4.87 × 10^−19^ J with the increase of electrostatic repulsion. The total interparticle potential energy of smithsonite is all negative when pH = 7. This illustrates the good dispersion behavior of smithsonite at pH = 4 and 10. There is cohesive behavior between particles at pH = 7, which, due to the agglomeration of particles, occurs about the IEP [28,29]. This is consistent with the results of turbidity tests and smithsonite surface zeta potential measurements.

In addition, the total potential energy of smithsonite in Figure 7 is negative at three pH conditions after the addition of Ca^2+^, which indicates that the particles produce coalescence and is consistent with the turbidity test results. When H < 17 nm, the interparticle interaction potential energy is higher at pH = 4. However, when H > 17 nm, the interparticle interaction potential energy at pH = 4 shows little change. At this time, the interparticle interaction potential energy at pH = 7 and 10 are more than pH = 4. This may be due to the CaOH^+^ or Ca(OH)_2(aq)_ component increase with the increasing pH, which leads to increased electrostatic repulsion between the particles.

### 2.6. Analysis of the Mechanism

**Figure 8:**
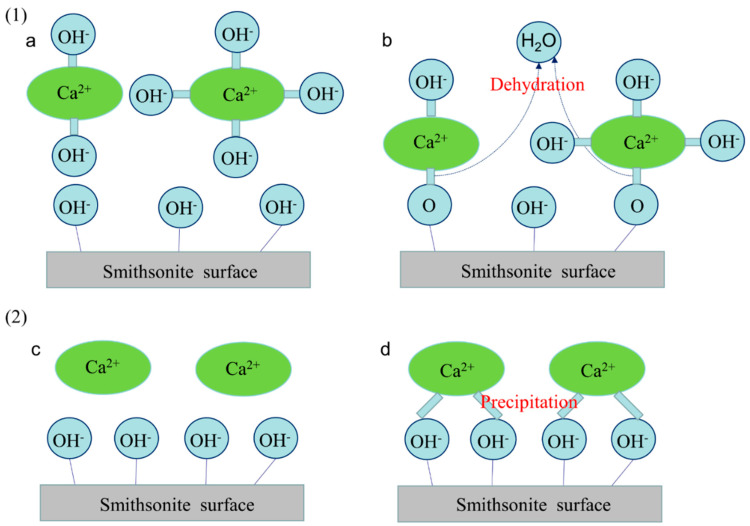
Mechanisms of metal ion action on mineral surfaces: (**1**) Formation of metal ion-hydroxy complexes on smithsonite surface: (**a**) before interaction; (**b**) after interaction. (**2**) Formation of metal hydroxide precipitation on smithsonite surface: (**c**) before interaction; (**d**) after interaction.

It is known from zeta potential and solution chemical analysis that calcium ions mainly compress the bilayer as the main factor at pH < 7, affecting the dispersion behavior of smithsonite. Ca(OH)^+^ and Ca(OH)_2_ are produced by the hydrolysis of calcium ions acting on the surface of smithsonite under alkaline conditions. At the same time, XPS analysis also showed that calcium hydroxide precipitation were generated out of the smithsonite surface, resulting in reduced interparticle forces. Therefore, it can be deduced that the mechanism of the interaction of calcium ions with the surface of smithsonite is as follows under alkaline conditions (pH = 8–12). Figure 8 shows that there are two main forms of metal ions acting on the mineral surface [30]. The first one is shown in Figure 8(1): calcium ions are dehydrated with OH^-^ in solution to form hydroxyl complexes, causing the formation of active sites on the smithsonite surface, which is conducive to collector action [30]. At the same time, the adsorption of calcium ions leads to changes in smithsonite surface potential, decreasing the potential energy of action on the smithsonite surface and leading to cohesive behavior between particles. Another form is shown in Figure 8(2): calcium ions form a precipitate on the smithsonite surface, which adheres or wraps around the smithsonite surface to change its physical and chemical properties more effectively [25].

## 3. Materials and Methods

### 3.1. Materials and Reagents

**Figure 9:**
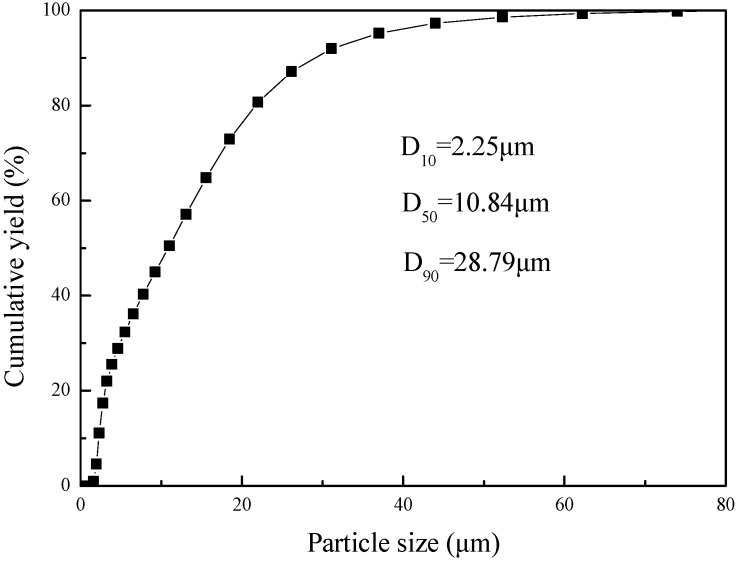
Particle size distribution of smithsonite.

Smithsonite mineral was produced from a zinc oxide mine in Yunnan Province, China. The D_10_, D_50_, and D_90_ of smithsonite particle sizes, as shown in Figure 9, were 2.25 μm, 10.84 μm, and 28.79 μm, respectively. Measurements were obtained by the S3500 laser particle size analyzer (Microtrac, Montgomeryville, PA, USA). Calcium chloride (CaCl_2_) was dissolved to prepare solutions of Ca^2+^ at predetermined concentrations. The reagents used in this study were all analytical grade. Deionized distilled water was used in all experiments, to eliminate the effect of ions in water on the dispersion behavior of smithsonite. Sampling using X-ray fluorescence (XRF) analysis was performed with an S8 Tiger (Bruker, Germany) instrument. The purity of the smithsonite was 97.01%, as shown in Table 3, which meets the test requirements.

### 3.2. Methods

#### 3.2.1. Turbidity Test

The test sample (1 g) and deionized water (40 mL) were placed together in a 100 mL beaker, and stirred for 5 min with a magnetic stirrer to fully disperse the sample. The dispersed slurry was poured into a 100 mL settling cylinder. The upper layer (25 mL) of the settling cylinder was measured using a turbidity meter 2100AN (HACH, Loveland, CO, USA).

#### 3.2.2. Zeta Potential Measurements

A sample of 20 mg (−5 μm) was taken and deionized distilled water (50 mL) was added; Ca^2+^ was then added to adjust the solution pH (4–12). The solution was stirred for 10 min with a magnetic stirrer, and left to stand for 12 h. The upper layer of the solution was taken to determine the zeta potential using the Zeta PALS system (Brookhaven, NY, USA).

#### 3.2.3. SEM and EDS Analysis

The samples of the turbidity test were vacuum dried and SEM and EDS analysis were performed using a Quanta 250 (FEI, Columbus, OH, USA). The surface of the test sample was gold plated and the mineral surface elemental analysis was performed in face analysis mode.

#### 3.2.4. XPS Analysis

The XPS used for the tests was an ESCALAB 250Xi from Thermo Fisher Scientific (Waltham, MA, USA). The sample (0.5 g) and deionized distilled water (50 mL) were placed together in a 100 mL beaker, and Ca^2+^ was then added to adjust the solution pH. The solution was stirred for 10 min with a magnetic stirrer and left to stand for 10 min, then the sample of filtration was vacuum dried to be XPS measured.

#### 3.2.5. DLVO Theoretical Calculation

To illustrate the mechanism of the influence of Ca^2+^ on smithsonite dispersion behavior, this study calculates the variation of interparticle forces in the different solutions of smithsonite according to DLVO theory. The following equation for the total energy of particles subjected to interaction in the DLVO theory is given [24,31]. In the medium, it is assumed that the particle shape is spherical, for the same mineral particle size R.

(12) $$V_{T}{=V}_{E}{+V}_{W}$$

(13) $$V_{W}=-\frac{A_{131}R}{12H}\;$$

(14) $$\;V_{E}{=2\pi \varepsilon }_{a}{R\psi }_{0}^{2}\ln\left[1+\exp\left(-\mathrm{kH}\right)\right]$$

where V_T_ is the total interparticle potential energy, J; V_E_ is the interparticle electrostatic potential energy, J; V_w_ is the potential energy of van der Waals forces, J; A_131_ is the Hamaker constant, J; R is the diameter of mineral particles, nm; H is the interparticle action distance (H < < R), nm; $\varepsilon_{a}$ is the absolute dielectric constant of the dispersed medium; R is the diameter of mineral particles, nm; ψ_0_ is mineral surface electric potential; k^−1^ is the Debye length, nm; and H is the interparticle action distance (H < < R), nm.

## 4. Conclusions

The dispersion behavior of smithsonite was better in the absence of calcium ions at pH = 4–12. The measured turbidity values of smithsonite decreased with the addition of calcium ions. In particular, the dispersion behavior of smithsonite was severely disrupted under strongly alkaline conditions. The analyses of Zeta potential and XPS, and calculations of solution chemistry, showed that calcium ions mainly compress the double layer effect to reduce the electrostatic potential energy between particles at pH < 7. However, calcium ions are adsorbed on the surface of smithsonite with the increase of pH, generating ion exchange or Ca(OH)^+^ and precipitation. In addition, the results of DLVO theory analysis showed that the potential barrier of smithsonite was about 15.54 × 10^−19^ J and 4.87 × 10^−19^ J at pH = 4 and 10, respectively. The total interparticle interaction potential energy was calculated to be negative in the presence of Ca^2+^, which destroyed the dispersion behavior of smithsonite.

## Figures and Tables

**Table 1:** The hydrolytic stability constants of Ca^2+^.

Metal Ions	α_1_	α_2_	α_3_	α_4_	K_S0_	K_S1_	K_S2_	K_S3_
Ca^2+^	1.4	2.77	/	/	5.22	3.82	/	/

**Table 2:** Atomic concentrations of main elements on smithsonite surfaces: (a) smithsonite (b) smithsonite with Ca^2+^.

Sample	Atomic Concentration, %			
	Zn2P	O1s	C1s	Ca2p
a	17.02	52.73	30.24	-
b	14.93	51.27	32.57	0.60

**Table 3:** XRF analysis spectra of smithsonite.

Composition	ZnCO_3_	Al_2_O_3_	SiO_2_	Fe_2_O_3_	Other	Total
Content (wt%)	97.01	0.26	0.89	0.22	1.62	100

## Data Availability

The data presented in this study are available in article.

## Sample Availability

Samples of the compounds are not available from the authors.
